# Depth dependence of climatic controls on soil microbial community activity and composition

**DOI:** 10.1038/s43705-021-00081-5

**Published:** 2021-12-15

**Authors:** Nicholas C. Dove, Morgan E. Barnes, Kimber Moreland, Robert C. Graham, Asmeret Asefaw Berhe, Stephen C. Hart

**Affiliations:** 1 Environmental Systems Graduate Group, University of California, Merced, CA 95343 USA; 2 Biosciences Division, Oak Ridge National Laboratory, Oak Ridge, TN 37830 USA; 3 Earth and Biological Sciences Division, Pacific Northwest National Laboratory, Richland, WA 99354 USA; 4 Center for Accelerator Mass Spectrometry, Lawrence Livermore National Laboratory, Livermore, CA USA; 5 Department of Environmental Sciences, University of California, Riverside, CA USA; 6 Department of Life & Environmental Sciences, University of California, Merced, CA 95343 USA; 7 Sierra Nevada Research Institute, Merced, CA 95343 USA

**Keywords:** Community ecology, Biogeochemistry

## Abstract

Subsoil microbiomes play important roles in soil carbon and nutrient cycling, yet our understanding of the controls on subsoil microbial communities is limited. Here, we investigated the direct (mean annual temperature and precipitation) and indirect (soil chemistry) effects of climate on microbiome composition and extracellular enzyme activity throughout the soil profile across two elevation-bioclimatic gradients in central California, USA. We found that microbiome composition changes and activity decreases with depth. Across these sites, the direct influence of climate on microbiome composition and activity was relatively lower at depth. Furthermore, we found that certain microbial taxa change in relative abundance over large temperature and precipitation gradients only in specific soil horizons, highlighting the depth dependence of the climatic controls on microbiome composition. Our finding that the direct impacts of climate are muted at depth suggests that deep soil microbiomes may lag in their acclimation to new temperatures with a changing climate.

## Introduction

Microbial community composition varies considerably throughout the soil profile [1] likely due to edaphic factors that change with depth, including organic carbon (C) availability, nutrients, pH, and texture [2–5]. However, most studies on subsoil microbial communities do not span large ecological gradients, preventing a conclusive understanding of large-scale drivers of subsoil microbial communities. Therefore, across broad ecological gradients, our understanding of the controls of subsoil microbial communities is inconclusive for bacteria [1] and even more limited for fungi. Climate is often considered a major driver of microbial community composition and activity at large spatial scales in the surface soil [6–8]. However, the role of climate in predicting subsoil microbial community composition and activity is still unclear. Given that subsoil microbial communities play a significant role in decomposing the ~1400 Pg of soil organic carbon (SOC) stored below 20 cm [9], understanding the large-scale controls over subsoil microbial communities is critical in predicting future soil C stocks and future temperatures.

The direct effects of climate on microorganisms are dictated by temperature, precipitation, and aridity, and how these factors vary and covary seasonally [10]. Given that microorganisms and their extracellular enzymes (EEs) have different temperature and moisture optima [11–13], climatic adaptations are common. Various microorganisms in the surface soil, particularly within the phyla Actinobacteria and Chloroflexi, have been shown to respond positively to increased temperatures [14]. Climate can also affect microbial communities indirectly through its influence on soil development. For instance, climate is one of the five state factors of soil development [15], affecting the availability of inorganic nutrients, soil texture, and organic matter [10, 16], all of which impact microbial community dynamics. Additionally, wetter climates generally lead to decreased pH [17], a major correlate of microbial community structure [18, 19], through base cation leaching and accumulation of iron and aluminum colloids [20]. Finally, climate often covaries with vegetation [21], leading to differences in organic substrate abundance and chemical composition, which also impacts microbial community composition and activity [22, 23]. These direct and indirect effects lead to strong bioclimatic patterns of soil microbial community structure and activity in surficial soils.

Climatic effects on subsoil microbial communities may differ compared to their surface soil counterparts. While the surface soil interfaces directly with air temperature and precipitation, the surface soil modulates the impacts of climatic changes on the underlying subsoil. Thus, seasonal and diurnal fluctuations in temperature and moisture are muted at depth [24, 25]. This could lead to two alternative scenarios: (1) subsoil microbial communities are *less* adapted to climate, whereby moderate temperature and moisture levels are not strong selective pressures; or (2) subsoil microbial communities are *more* adapted to climate, whereby reduced temperature and moisture fluctuations select for highly constrained microbial communities. Recent evidence suggests that subsoil microbial community composition may be less responsive to climatic conditions. For example, the effect of 4.5 years of experimentally increased temperature on microbial community composition and metabolism was reduced at depth. This was likely due to metabolic capabilities of subsoil microbial communities that allow for the decomposition of a variety of complex organic matter substrates that are enriched with warming [26]. Additionally, laboratory warming (+10 °C) of Tibetan soils showed that subsoil microbial communities are, in general, less responsive to altered temperatures, at least in the short-term (30 days) [27]. However, the full effect of climate (i.e., including how climate affects biota and soil formation) may not emerge over such short timescales, because the indirect effects of climate (i.e., changes in vegetation and soil chemistry) may take decades to develop. Therefore, well-constrained, observational studies investigating the longer-term direct and indirect effects of climate on the subsoil microbial community are particularly valuable for capturing the full effect of climate.

Understanding the controls on microbial communities at depth is particularly important because they are relatively understudied [28] and they play a significant role in decomposing the ~1400 Pg of SOC stored below 20 cm [9]. For instance, over half of EE activity in the upper meter of the soil profile occurs below 20-cm [5]. Furthermore, when warmed, subsoil respiration can account for over 40% of the increase in CO_2_ emissions from the whole soil profile [29]. Finally, laboratory rates of C and nitrogen (N) mineralization of added substrates were as fast in the subsoils as in surface soils in an old-growth forest, suggesting that microbial competition and demand for C and N resources does not decrease with depth [30]. Taken together, these results suggest that subsoil microbial communities are important mediators of deep C persistence. Within the context of a changing climate, climate-induced alterations to the microbial community could enhance SOC loss from the subsoil, exacerbating climate change [31, 32]. Hence, our understanding of the climatic controls of subsoil microbial communities and how these differ from surface soils is integral in constraining long-term soil C storage predictions.

**Fig. 1:**
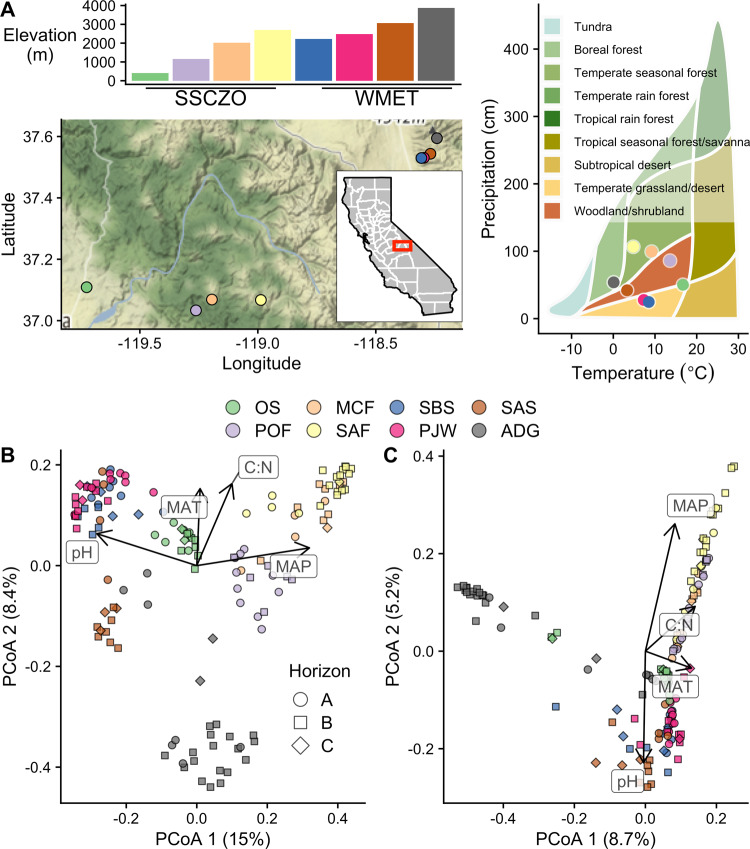
Overview of the study sites. Elevation, location, and biome (note: while ADG is classified as a boreal forest, it is an alpine tundra) are shown (**A**). Principal coordinates analysis (PCoA) plots of prokaryote (**B**) and fungal (**C**) community composition. The percentage in parentheses indicates the variation explained by each axis. Vectors represent the direction and magnitude (indicated by vector length) of correlations of environmental variables with the first two axes of the PCoA. Key: MAP mean annual precipitation, MAT mean annual temperature, C:N the soil carbon-to-nitrogen ratio (molar basis).

To understand the impact of climate on microbial community composition and activity throughout the soil profile, we collected soil samples from each genetic horizon (A–C) in soil profiles along two elevational gradients (Fig. 1A). Elevational transects are valuable bioclimatic gradients that can be used to study the long-term impact of climate on soils, particularly when other soil development state factors are held constant [33]. These samples were analyzed for prokaryote and fungal community composition (16S rRNA and ITS gene, respectively), potential activity (using EE activity assays), and soil chemistry. We hypothesized that the microbial community composition would vary among sites and with depth (in cm, herein referred to as “depth”) or soil master horizon. Given our study design, we were able to test the hypothesis that soil master horizon would be a stronger predictor of microbial community composition than depth. Additionally, we hypothesized that, the direct effect of climate on microbial community composition and activity would be reduced at depth, consistent with the few previous studies conducted to date (e.g., Bai et al. [27], Dove et al. [26]). Deeper in the soil profile, the *indirect* effects of climate, namely soil chemistry, would become the dominant control of microbial community composition and activity. We also hypothesized that certain microbial taxa would correlate with the direct effects of climate both throughout the soil profile and within master horizons based on their taxonomically inferred metabolism. The overall goal of our study was to provide a greater understanding of microbial communities with depth and to elucidate their patterns as they relate to climate. Such knowledge should improve our ability to predict microbial decomposition and soil C stocks in a changing climate.

## Methods

### Study sites

We sampled soils at eight sites along two bioclimatic gradients based on elevational transects in Central California, USA (Fig. 1A). The Southern Sierra Critical Zone Observatory (SSCZO) experiences a Mediterranean-type climate that traverses the west side of the Sierra Nevada [34]. From west to east, sites increase in elevation, increase in precipitation, and decrease in temperature, and they are defined as follows: oak savannah (OS), pine-oak forest (POF), mixed conifer forest (MCF), and subalpine forest (SAF; Fig. 1A). The White Mountain Elevational Transect (WMET) experiences a semiarid-type climate (i.e., significantly drier than SSCZO, Fig. 1A) along the west side of the White-Inyo Range [35]. From west to east, sites also increase in elevation, increase in precipitation, and decrease in temperature and are defined as follows: sagebrush shrubland (SBS), pinyon-juniper woodland (PJW), subalpine shrubland (SAS), and alpine desert grassland (ADG; Fig. 1A; see Table S1 for further site characteristics). Soil age across our sites is difficult to determine because of significant aeolian and colluvial deposits [36, 37]; however, all soils are derived from granite, granodiorite, or tonalite, and only SAF was glaciated [34, 35]. Therefore, we contend that bioclimatic influences dominated differences in soils across the eight sites.

### Sample collection

Sampling occurred during the dry seasons (late summer, early fall) of 2014 and 2015. Because the microbial communities in these climates are moisture-limited, the microbial communities at these sites are relatively dormant during the dry season—EE activities during the summer/fall are about a third of those in the spring [38]. While overall EE activities may be lower, sampling during the dry season minimizes the temporal effects of sampling at different times. Four soil profiles were dug at each site at least 20 m apart representing the major topographic features of the landscape (i.e., stratified sampling). To minimize the effect of plant roots, all profiles in the SSCZO were dug under relatively open canopies, and within hours after excavation, soil was collected based on master and subordinate genetic horizons (Table S2). Soil genetic horizons were classified following the USDA NRCS taxonomic system [39]. At WMET sites, SBS, PJW, and SAS, there are considerable soil chemical differences between areas under shrubs and the interspace [35]. We sampled only under shrubs to keep sampling consistent with other sites that did not have interspace components. Sampling tools were sanitized with 10% bleach followed by 70% ethanol and were used to dig horizontally into a single soil profile face (after clearing away exposed soil). Soil profiles were 1 m wide, and we sampled across the horizon. Hence, replication for all analyses was assessed at the soil profile level (*n* = 4). Soils were immediately placed on dry ice for transport back to the laboratory.

### Soil chemistry

Soil samples were air dried and sieved (<2 mm) for chemical analyses. We determined elemental concentrations of iron, aluminum, calcium, phosphorus (P), potassium, magnesium, and silicon using lithium metaborate fusion [40] measured by inductively-coupled plasma optical-emission spectrometry (ICP-OES; Perkin-Elmer Optima 5300 DV; University of California, Merced Environmental Analytical Laboratory). We measured organic C and total N on an elemental analyzer (Costech Analytical ECS 4010 Elemental Analyzer, Costech Analytical Technologies, Inc., Valencia, CA; Stable Isotope Laboratory at the University of California, Merced). Carbonates were removed from WMET sites prior to analysis of organic C using hydrochloric acid fumigation [41] (no effervescence occurred after 1 M HCl addition to SSCZO sites, so organic C was considered equivalent to total C). Soil pH was measured in a 1:2 suspension:solution w/v in deionized water and 0.01 M CaCl_2_ (Accumet Basic, Model AB15, Fisher Scientific with an Ag/AgCl electrode; [42]).

### Microbial biomass and extracellular enzyme activity

We measured microbial biomass C (MBC) by chloroform fumigation-extraction using 10–50 g (depending on sample weight availability and master horizon—greater soil weight was used in deeper horizons) of previously frozen and thawed, field-moist mineral soil [43–45]. Performing chloroform fumigation-extraction on previously frozen soils may slightly affect the absolute values, but the relatively differences among treatments are generally unaffected [46].

We measured potential EE activity (i.e., activity not limited by substrate concentrations) of β-glucosidase (BG), N-acetylglucosaminidase (NAG), and acid phosphatase (AP) fluorometrically following Bell et al. [47]. These three enzymes are considered C-, N-, and P-acquiring enzymes, respectively. EE activities were expressed per soil weight (mmol EE activity kg^−1^ soil h^−1^), SOC (mmol EE activity kg^−1^ SOC h^−1^), and MBC (mmol EE activity kg^−1^ MBC h^−1^). These latter two variables are called SOC-normalized and MBC-normalized, respectively in this paper.

### DNA extraction

We extracted total soil DNA using the MoBio PowerSoil DNA isolation kit (Carlsbad, CA) following the manufacturer’s instructions. We extracted DNA from 1 g of soil using four parallel, replicate extractions (0.25 g per extraction), and replicate extracts were pooled onto a single column and eluted in 50 μL for downstream analysis to increase DNA yield. We quantified DNA yields using the Quant-it PicoGreen dsDNA assay kit (Invitrogen, Carlsbad, CA).

### PCR amplification, sequencing, and bioinformatics

Sample libraries were prepared and sequenced at the Environmental Sample Preparation and Sequencing Facility at Argonne National Laboratory (Lemont, IL). For prokaryotes (archaea and bacteria), 16S rRNA genes were amplified in PCRs using primers (515F/806R) that target the V4 region of the 16S rRNA gene [48]; for fungi, ITS2 regions were amplified in PCR reactions using ITS9f/ITS4R primers [49, 50]. Each 25 µL PCR reaction contained 9.5 µL of MoBio PCR Water (Certified DNA-Free), 12.5 µL of QuantaBio’s AccuStart II PCR ToughMix (2x concentration, 1× final), 1 µL Golay barcode tagged forward primer (5 µM concentration, 200 pM final), 1 µL reverse primer (5 µM concentration, 200 pM final), and 1 µL of template DNA. The conditions for PCR are as follows: 94 °C for 3 minutes to denature the DNA, with 35 cycles at 94 °C for 45 s, 50 °C for 60 s, and 72 °C for 90 s; with a final extension of 10 min at 72 °C to ensure complete amplification. Amplicons were then quantified using PicoGreen (Invitrogen, Carlsbad, CA) and a plate reader (Infinite® 200 PRO, Tecan Group Ltd., Männedorf, Switzerland). Once quantified, volumes of each of the products were pooled into a single tube in equimolar amounts. This pool was then cleaned using AMPure XP Beads (Beckman Coulter) and quantified using a fluorometer (Qubit, Invitrogen, Carlsbad, CA). After quantification, the molarity of the pool was determined, diluted down to 2 nM, denatured, and then diluted to a final concentration of 6.75 pM with a 10% PhiX spike for sequencing on the Illumina MiSeq platform (Illumina Inc., San Diego, CA), resulting in 251 bp paired-end reads.

Both 16S and ITS2 datasets were denoised, joined, delineated into amplicon sequence variants (ASVs) using DADA2 [51], without further trimming/truncation prior to ASV delineation in the QIIME2 environment (v. 2019.7; [52]). We then assigned representative sequences a taxonomic classification using the Naïve Bayes classifier through the sklearn python package for 16S rRNA sequences with the SILVA database (Release 132; [53]) and a confidence of 0.7. We assigned taxonomic classifications of the ITS2 of the ribosomal operon to representative sequences using consensus BLAST (% identity = 80%, *e* value = 0.001, and minimum fraction of assignments = 0.51; [54]) and the UNITE reference database (version 8.0, [55]). Fungal ASVs were further classified as ectomycorrhizal (EM) using FUNGuild [56]; all ASVs assigned to the family Glomeraceae were classified as arbuscular mycorrhizal (AM) fungi. All sequence data are deposited at the Sequence Read Archive under the Bioproject PRJNA743681.

### Statistical analysis

All statistical analyses were conducted in R v. 4.0.2 [57], with the betareg [58], car [59], lme4 [60], phyloseq [61], and vegan [62] packages. For all statistical tests, significance was defined at the α = 0.05 level. The R code used to conduct statistical analyses and generate figures can be found at https://github.com/nicholascdove/climate_subsoil_microbiome.

Differences in the community composition of the prokaryotes and fungi among sites and with depth or by horizon were assessed by PERMANOVA [63]. Multiple models were conducted to investigate how community composition is impacted at all sites (Full Model), or the SSCZO and WMET separately. For these models, site identity as well as depth or master horizon were used as independent variables. Differences in beta-diversity among soil master horizons as well as overall heterogeneity of multivariate dispersions were tested by using the ‘betadisper’ function in vegan [62, 64]. Prokaryote and fungal community compositions were visualized using principal coordinates analysis (PCoA). Environmental vectors were fit onto PCoA ordinations using the ‘envfit’ function in vegan. For the PERMANOVAs, beta-diversity, and PCoAs, we used Bray-Curtis dissimilarity applied to proportionally normalized data.

The predictive power of depth (in cm) and master horizon in explaining microbial community composition were compared by first assessing the significance of “depth” or “horizon” in the aforementioned PERMANOVA models. If both were significant, then the R^2^ value of the “depth” or “horizon” term was assessed, and terms with higher R^2^ values were considered to be better predictors.

The relative control of the direct effects of climate (i.e., MAP and MAT) versus the indirect effects of climate (i.e., soil chemistry) on microbial community composition was assessed using variance partitioning [65], again using Bray-Curtis dissimilarity applied to proportionally normalized data. For these variance partitioning models, the effect of MAT and MAP were combined to form the variable “climate,” and the effect of pH as well as the effect of the molar concentrations of the aforementioned elements were combined to form the variable “soil chemistry.” This resulted in variance partitioning models with three independent variables: climate, soil chemistry, and their interaction. The significance of variables was determined using ANOVA and distance-based redundancy analysis (db-RDA, [66]), sequentially “partialling out” variables.

To determine the impact of MAT and MAP with depth, multiple mixed effects models were fit to our EE activities with the fixed effects of depth, MAT and MAP and the random effect of pit. These *a priori* defined models are as follows: (1) log_10_(enzyme) ~ Depth + (1|Pit), (2) log_10_(enzyme) ~ Depth + MAT + Depth × MAT + (1|Pit), (3) log_10_(enzyme) ~ Depth + MAP + Depth × MAP + (1|Pit), and (4) log_10_(enzyme) ~ Depth + MAT + Depth × MAT + MAP + Depth × MAP + (1|Pit). Enzyme activities were log_10_ transformed to satisfy assumptions of normality and homoscedasticity.

Significant changes in the relative abundance of individual major microbial taxa with depth, MAT, and MAP were determined by analysis of compositions of microbiomes with bias correction (ANCOM-BC [67]). For differences in mycorrhizal relative abundance among sites and master horizons, we used beta regression, which fit the distributions of the dependent variables (i.e., percentages). Where significant, multiple comparisons among sites were assessed by Tukey’s HSD.

## Results

### Site identity explains greater variation in microbial community composition than depth

While both site identity and depth were significant moderators of microbial community composition, site identity generally explained a greater amount of the variation in the microbial community composition than depth, likely due to differences in climate, soil chemistry, and vegetation (Fig. 1; Table 1). However, there was a significant site identity by depth interaction on microbial community composition, such that depth did not significantly affect microbial community composition at all sites (Fig. S1, Table 1). For prokaryotes, depth was not a significant moderators of community composition at MCF and POF (Table 1), two of the three wettest sites in our study (Fig. 1A), and for fungi, depth was only a significant moderator of community composition at ADG, SBS, and SAF (Table 1).

**Table 1:** The *R*^2^ of “horizon” or “depth” in prokaryote and fungi PERMANOVA models when either was included.

	Prokaryote					Fungi				
Model	Site	Horizon	Depth	Site × Horizon	Site × Depth	Site	Horizon	Depth	Site × Horizon	Site × Depth
Full	**0.346**	**0.039**	**0.024**	**0.122**	**0.089**	**0.221**	**0.021**	**0.010**	**0.109**	**0.068**
SSCZO	**0.278**	**0.073**	**0.045**	**0.104**	**0.069**	**0.143**	**0.036**	**0.019**	**0.086**	**0.061**
OS		**0.255**	**0.164**				0.167	0.089		
POF		**0.156**	0.137				0.096	0.096		
MCF		0.189	0.126				0.160	0.072		
SAF		**0.375**	**0.201**				0.145	**0.110**		
WMET	**0.255**	**0.082**	**0.051**	**0.102**	**0.085**	**0.218**	**0.049**	**0.024**	**0.101**	**0.061**
SBS		**0.212**	**0.155**				0.205	**0.124**		
PJW		**0.238**	**0.195**				0.147	0.110		
SAS		**0.387**	**0.206**				0.249	0.118		
ADG		**0.218**	**0.182**				**0.296**	**0.091**		

The Full, SSCZO, and WMET models also included “site” and the interaction of “horizon” and “site” or “depth” and “site” as factors. Bolded values indicate significance of the factor (*p* < 0.05).

Master horizon was also a significant factor affecting microbial community composition. While the variation in the microbial community explained by horizon was overall lower than site identity, master horizon consistently explained a greater amount of the variation in microbial community composition than depth (Table 1). In fact, horizon was a significant moderator of prokaryote community composition in all sites except MCF (Table 1). However, horizon was only a significant moderator of fungal community composition at ADG (Table 1). On average, across all PERMANOVAs, horizon explained 51% and 89% more variation than depth for prokaryote and fungal community composition, respectively (Table 1).

Among master horizons, prokaryote beta-diversity was similar across the entire dataset (ANOVA: *F*_2,141_ = 1.6, *p* = 0.215, Fig. S2). However, fungal beta-diversity significantly differed by horizon (*F*_2,143_ = 3.8, *p* = 0.026), with the A horizon having a significantly higher beta-diversity than the B horizon (Tukey HSD: *p* = 0.032).

### The direct effect of climate on the microbial community declines with depth

**Fig. 2:**
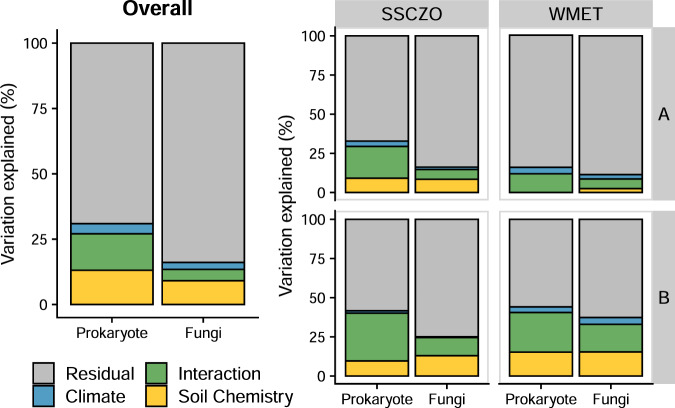
Variance partitioning from the PERMANOVA of prokaryote and fungal community composition, overall, across elevational gradients, and among master horizons (lack of samples in the C horizon prevented analysis in this layer). Climate is the combined effect of mean annual temperature and precipitation. Soil chemistry is the combined effect of pH (1:2 CaCl_2_) as well as the concentration of total extractable aluminum, calcium, carbon, iron, magnesium, nitrogen, phosphorus, potassium, silicon, and sodium (mmol kg^−1^).

Across elevational gradients and master horizons, both climatic factors (i.e., MAT and MAP) and soil chemistry (i.e., pH and elemental concentrations) significantly influenced prokaryote (soil chemistry: *F*_11,139_ = 3.4, *p* < 0.001; climate: *F*_2,139_ = 4.6, *p* < 0.001) and fungal community composition (soil chemistry: *F*_11,141_ = 2.4, *p* < 0.001; climate: *F*_2,141_ = 3.1, *p* < 0.001; Fig. 2A). However, the variance explained by soil chemistry was almost three and a half times greater than that of climate. Within each elevational gradient, the relative effect of climate (compared to soil chemistry) decreased with depth (Fig. 2B). For prokaryotes, the effect of climate was minimal in the B horizon at the SSCZO, as was the effect of soil chemistry in the A horizon at WMET. For fungi, while the effect of soil chemistry on community composition was relatively similar to that of climate in the A horizon; soil chemistry explained three to seven times more variance in the composition than climate in the B horizon.

### The impact of climate on extracellular enzyme activity depends on normalization method and depth

EE activity per unit soil weight consistently decreased with soil depth (Fig. S3). Still, when normalized by mol MBC or SOC, EE activity *increased* with soil depth (Table 2). Per unit soil weight, EE activity was generally unrelated to climatic factors when controlling for soil depth (Table 2). However, normalized by mol MBC, the activities of NAG and AP were positively influenced by increasing MAT and MAP, and normalized by SOC, the activities of NAG and AP were positively influenced by increasing MAT (Table 2). However, the effect of these climate variables interacted with soil depth, where the effect of both MAP and MAT diminished with increasing soil depth (Table 2). For BG per mol MBC, there was only a main, positive effect of MAP, which interacted negatively with soil depth; BG activity per mol SOC was not influenced by the main effects of MAT and MAP. However, the effect of soil depth on BG activity per mol MBC decreased with increasing MAP (Table 2).

**Table 2:** Coefficients of competing mixed effects models of enzyme activity (μmol) normalized by soil weight, microbial biomass carbon (MBC), and soil organic carbon (SOC).

	Enzyme	Model^a^	Depth	MAT	Depth × MAT	MAP	Depth × MAP
μmol kg^−1^ soil	BG	1	**−3.43E**−**03**				
		2	**−6.21E**−**03**	−2.13E−02	2.74E−04		
		3	−6.28E−04			2.11E−04	−3.84E−06
		4	−3.90E−03	−2.11E−02	2.55E−04	2.09E−04	−2.96E−06
	NAG	1	**−2.60E**−**03**				
		2	−1.70E−03	1.58E−02	−1.08E−04		
		3	1.64E−03			5.15E−04	−6.05E−06
		4	3.56E−03	1.90E−02	−1.57E−04	**5.45E−04**	−6.71E−06
	AP	1	**−1.89E**−**03**				
		2	−2.28E−03	1.02E−02	1.87E−05		
		3	2.00E−03			2.61E−04	−5.29E−06
		4	2.00E−03	1.09E−02	−2.36E−05	2.46E−04	−5.16E−06
μmol mol^−1^ MBC	BG	1	3.09E−03				
		2	**9.39E**−**03**	3.13E−02	**−7.98E−04**		
		3	**1.62E**−**02**			**9.42E−04**	**−1.67E−05**
		4	**1.81E**−**02**	2.15E−02	−6.06E−04	**8.67E−04**	−1.33E−05
	NAG	1	1.86E−03				
		2	**1.31E**−**02**	**8.50E−02**	**−1.40E−03**		
		3	**2.17E**−**02**			**1.44E−03**	**−2.45E−05**
		4	**2.66E**−**02**	**7.70E−02**	**−1.21E−03**	**1.30E−03**	**−1.89E−05**
	AP	1	**5.14E**−**03**				
		2	**1.39E**−**02**	**6.80E−02**	**−1.14E−03**		
		3	**2.21E**−**02**			**9.88E−04**	**−2.14E−05**
		4	**2.47E−02**	**5.80E−02**	**−8.78E−04**	**7.86E−04**	**−1.63E−05**
μmol mol^−1^ SOC	BG	1	**4.59E−03**				
		2	**5.92E−03**	−1.74E−03	−1.13E−04		
		3	**1.47E−02**			−7.21E−05	**−1.27E−05**
		4	**1.79E−02**	5.76E−03	−2.32E−04	−6.86E−05	**−1.35E−05**
	NAG	1	**4.43E−03**				
		2	**9.67E−03**	**4.64E−02**	**−5.21E−04**		
		3	**1.54E−02**			2.67E−04	**−1.42E−05**
		4	**2.49E−02**	**5.56E−02**	**−7.05E−04**	3.63E−04	**−1.73E−05**
	AP	1	**6.40E−03**				
		2	**1.07E−02**	**3.03E−02**	**−4.21E−04**		
		3	**1.68E−02**			−6.64E−05	**−1.32E−05**
		4	**2.29E−02**	**3.50E−02**	**−1.43E−05**	−8.81E−05	**−1.43E−05**

Bolded coefficients represent significant (*p* < 0.05) fixed effects.

*BG* β-glucosidase, *NAG* N-acetylglucosamine, *AP* acid phosphatase, *MAT* mean annual air temperature (°C), *MAP* mean annual precipitation (mm).

^a^1 = log_10_(enzyme) ~ Depth + (1|Pit)

2 = log_10_(enzyme) ~ Depth + MAT + Depth × MAT + (1|Pit)

3 = log_10_(enzyme) ~ Depth + MAP + Depth × MAP + (1|Pit)

4 = log_10_(enzyme) ~ Depth + MAT + Depth × MAT + MAP + Depth × MAP + (1|Pit).

Substituting the effect of master horizon for the effect of depth on EE activities revealed similar patterns, with master horizon being a strong moderator of EE activity (Table S3). However, for BG and NAG activities (across all normalization methods), the effect of horizon on activity consistently interacted with MAT. Similar to models including soil depth, the effect of MAT on EE activity decreased in subsoil horizons (Figs. S4–S6).

### Response of specific taxa to climate and depth

**Fig. 3:**
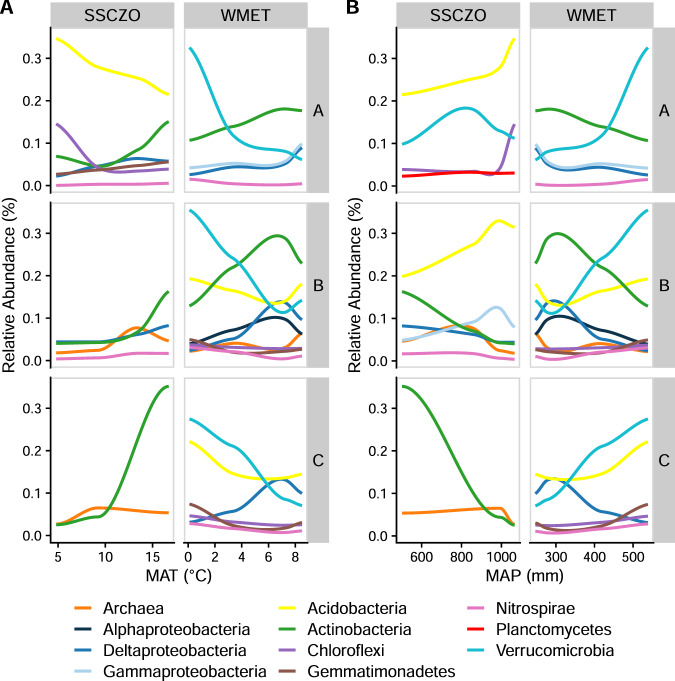
Differentially abundant prokaryotic taxa. Average relative abundance of prokaryotic taxa that significantly change (*p*-adjusted < 0.05) with mean annual air temperature (MAT, **A**) and mean annual precipitation (MAP, **B**) within each horizon and elevational gradient. Lines are calculated using loess regression of average relative abundance at each site (*n* = 4).

**Fig. 4:**
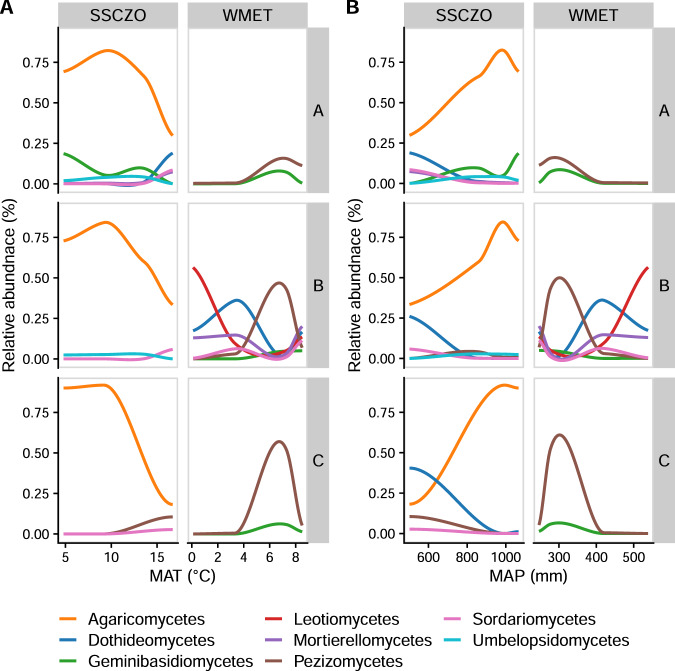
Differentially abundant fungal classes. Average relative abundance of fungal classes that significantly change (*p*-adjusted < 0.05) with mean annual temperature (MAT, **A**) and mean annual precipitation (MAP, **B**) within each horizon and elevational gradient. Lines are calculated using loess regression of average relative abundance at each site (*n* = 4).

Overall, the number of differentially abundant taxa were similar (range: 2–9) across elevation gradients and master horizons (Figs. 3 and 4). However, certain microbial taxa responded consistently to differences in MAT and MAP, while others followed idiosyncratic patterns across elevation gradients and master horizons (Figs. 3 and 4). For example, the relative abundance of Actinobacteria and Deltaproteobacteria consistently responded positively to increasing MAT, and the relative abundance of Acidobacteria consistently responded positively to increasing MAP (Fig. 3). However, the relative abundance of Agaricomycetes was positively correlated with MAP and negatively correlated with MAT only in the SSCZO gradient (Fig. 4). The same was also true for Verrucomicrobia, but only in the WMET gradient (Fig. 3). On the other hand, the relative abundance of Archaea was negatively correlated with MAP and correlated positively with MAT, but only in subsurface soil horizons across both gradients (Fig. 3).

The relative abundances of many microbial taxa were also significantly impacted by depth, but only in certain sites (Fig. S7). For example, the relative abundance of Chloroflexi and Dothideomycetes increased with depth in only two of the eight sites (MCF and ADG for Chloroflexi and OS and SAF for Dothideomycetes, Fig. S7). Alternatively, the relative abundance of Agaricomycetes and Alphaproteobacteria decreased with depth in only three (SBS, SAS, ADG) and four sites (OS, POF, SAF, ADG), respectively (Fig. S7). The relative abundance of Bacteroidetes consistently decreased with depth, while other taxa had idiosyncratic depth distributions depending on the site (Fig. S7). At the two wettest sites, the relative abundance of Peziziomycetes decreased with depth, and in a single site in the WMET gradient (PJW) the relative abundance of Pezizomycetes increased with depth. Similarly, the relative abundance of Actinobacteria decreased with depth in the wettest site and increased with depth in the three driest sites (Fig. S7).

The influence of depth on the relative abundance of EM and arbuscular mycorrhizal (AM) fungi interacted with site (EM: *F*_7,143_ = 6.1, *p* < 0.001, AM: *F*_7,143_ = 2.1, *p* = 0.041), such that depth was a significant moderator of EM fungi only at SH (increased) and of AM fungi at SBS and ADG (decreased; Fig. S8, Table S4). However, the patterns of EM and AM relative abundances among sites were generally consistent among horizons. The relative abundances of EM fungi were generally higher in forested ecosystems such as POF, MCF, SAF, and PJW. In comparison, the relative abundances of AM fungi were generally higher in grassland ecosystems, such as OS, SBS, and SAS (Fig. S8).

## Discussion

It is becoming increasingly evident that microorganisms in the subsoil play important roles in soil C and nutrient cycling [5, 26, 29, 31], yet our understanding on the controls of microbial communities in the subsoil is limited. Here, we not only show that microbial community composition changes with depth, but also that the impact of climate, which strongly controls the surface soil microbial community composition, decreases in subsoil horizons [7, 8, 68, 69]. Furthermore, we find that certain microbial taxa change in relative abundance over large temperature and precipitation gradients only in specific soil horizons, highlighting the depth dependence of the climatic controls on microbial community composition.

Consistent with our hypothesis, the indirect effects of climate (i.e., soil chemical properties) became a more dominant control of microbial community composition with soil depth. At depth, seasonal and diurnal fluctuations in temperature and moisture are muted [24, 25], such that temperature and moisture extremes common in the surface that would likely select for certain microbial taxa [70, 71] may not occur in the subsoil. Instead, edaphic factors such as organic C limitation and changes in soil nutrients with depth [4, 9, 72] were apparently stronger moderators of subsoil microbial community composition. Our results support this assertion as oligotrophic and lithotrophic microorganisms adapted to low organic C availabilities increased in relative abundance with depth. For example, Bacteroidetes, which are generally considered copiotrophic [73], decreased in relative abundance with soil depth, while Nitrospirae and Archaea, which are commonly lithotrophic [74], increased in relative abundance with soil depth. However, we recognize that climate and soil chemistry together only explained a fraction of the microbial community composition (Fig. 2). This could be due to unmeasured environmental factors, such as soil water potential; biotic interactions among microbes, mesofauna, and viruses; or stochastic assembly processes [75]. Nevertheless, conceptually, we propose that the direct influence of climate extends from the atmosphere into the surface while the subsoil is influenced by the longer-term effects of climate, namely soil chemistry. This suggests that extrapolating microbial community composition based solely on climate variables when the climate is changing may not be appropriate in deeper soil layers.

While the effect of climate on microbial communities diminished in the subsoil, there were still microbial taxa that consistently responded to changes in climate across soil horizons. For instance, Actinobacteria consistently increased in relative abundance with increasing temperatures. This is consistent with previous research in the surface soil showing Actinobacteria phylotypes have greater relative abundances at warmer sites and respond positively to increasing temperature in laboratory incubations [14]. Furthermore, in field warming studies, Actinobacteria also increase in relative abundance in the surface [76, 77] and subsoil [26] in response to warming. In contrast, Archaea generally increased in relative abundance with increasing temperatures only in the subsoil. The relative abundance of Archaea does not generally correlate positively with temperature in the surface soil [78], but they have been shown to increase in relative abundances with permafrost warming in deeper layers [79]. Our results show that Archaea respond positively to temperature at depth in temperate forests as well. However, it is important to recognize that differences in relative abundances may not reflect changes in total abundance. We were unable to correct our relative abundances through qPCR using 16S and ITS primers because 16 S and ITS copy numbers in microbial genomes vary by an order of magnitude [80], and therefore, this analysis does not accurately represent microbial abundances over large differences in microbial community composition (as was the case in this study). Thus, our results suggest that characterizing responses of the relative dominance of microbial taxa to temperature is depth-dependent, underscoring the need of whole profile warming studies to elucidate the response of microbial communities to increased temperatures (e.g., Johnston et al. [79], Dove et al. [26]).

The depth dependence of climate impacts on microbial community composition also extended to microbial activity. We found that the impact of depth on microbial activity interacted with climate such that the positive effect of MAT and MAP on MBC- and SOC-normalized EE activity was muted in deeper soil horizons. This suggests that while EE activity on a MBC or SOC basis is positively correlated with MAT and MAP [6], this may not be the case in deeper soils. It is possible that greater clay stabilization of EE activities at depth may have reduced the influence of climate on microbial activity because higher microbial activities would reflect soil mineralogy rather than biology [5, 81, 82]. Hence, our results provide another line of evidence showing that the direct effects of climate (i.e., MAT and MAP) on microbial communities diminish with depth.

It was somewhat surprising that site identity explained over an order of magnitude more variation in microbial community composition than depth because many physical and chemical properties of soil change dramatically throughout the soil profile. For instance, organic C concentration decreases with increasing soil depth [4], selecting for microorganisms capable of utilizing a myriad of substrates [26] or even alternative energy sources, such as CO [1]. Hence, the effect of soil depth on microbial community composition has been shown to be as strong as the effect of location [83]. However, over relatively broader ecological gradients, the effect of site identity can become stronger than soil depth [1]. This discrepancy is probably the result of greater soil chemical and climatic heterogeneity across sites, which likely explains the findings in this study as our sites spanned multiple bioclimatic envelopes (Fig. 1A) and included large differences in soil chemistry. For example, the relative abundance of EM and AM largely correlated with whether the ecosystem was treE− or grass-dominated and was generally not influenced by depth. It is also possible that differences in the maximum depth of the soil could have contributed to site specific differences in microbial community composition. For instance, microbial dispersal limitations may increase with greater depth, decreasing the available species pool in deeper soil layers. Thus, deeper soils could result in greater microbial isolation affecting community assembly. Also, if the lateral movement of soil microbes across large distances occurs primarily aboveground [84, 85], then the species pool for subsurface colonization is constrained by surface soil conditions. In other words, subsoil microbial communities need to pass through two environmental filters: the condition of the surface soil and the subsoil counterpart. Indeed, in a temperate grassland ecosystem, dispersal limitation across 2 km and selective determinism was found to increase with soil depth [86]. In the context of a changing climate, this apparent dispersal limitation suggests that changes to the community composition of the subsoil will likely follow that of the surface soil.

Our finding that master soil genetic horizon explained a greater proportion of the variation in microbial community composition than depth suggests that depth-resolved microbial ecology should sample based on master or subordinate genetic horizons when possible. Commonly, soils are sampled by depth rather than master horizon [1, 5, 26], because delineating genetic horizons necessitates an in-depth understanding of physical and chemical nature of the soil profile and technical expertise. Furthermore, soil characterization is often only possible through full soil profile excavation rather than deep soil cores sampled from the surface (i.e., how deeper soil layers are commonly sampled in microbial ecology). Because we found that soil chemistry is a strong moderator of microbial community composition and genetic horizons are in part delineated by soil chemistry [87], it is unsurprising that genetic horizons explained a greater proportion of the variation in microbial community composition than depth. It is also possible that genetic horizon explained a greater proportion of the variation than depth because, unlike depth, horizon is a categorical variable. Categorical variables can better describe often observed nonmonotonic changes in the relative abundance of microbial taxa with depth (Fig. S7), whereas continuous variables better describe linear patterns.

The differences in climate exhibited in this study are relatively greater than what is expected within the next century due to climate change [88]. However, these observational findings corroborate recent experimental findings suggesting that the microbial response to increased temperatures is subdued at depth [26]. Additionally, unlike experimental warming, these results incorporate the longer-term nature of climate effects, including both the direct and indirect (i.e., changes in soil chemistry and vegetation) effects of increased temperatures. Bradford et al. [89] proposed that microbial temperature acclimation might mitigate enhanced microbial respiration due to increased temperatures with climate change. However, our main finding that the direct impacts of climate are reduced at depth suggests that deep soil microbial communities may lag in their acclimation to new temperatures, potentially allowing for continued enhanced microbial respiration rates that further increase atmospheric CO_2_ levels.

## Supplementary information

Supplementary Material

Supplementary Material

## Data Availability

All sequence data can be accessed through the sequence read archive under Bioproject PRJNA743681. Soil chemistry and extracellular enzyme data is archived in the Dryad repository (10.6071/M3XM3S).
