# Supplementary material for: Depth dependence of climatic controls on soil microbial community activity and composition
Source: ISME Commun. 2021 Dec 15;1:78. doi: 10.1038/s43705-021-00081-5 (PMC9723684; doi:10.1038/s43705-021-00081-5)

Supplemental information for "**Depth dependence of climatic controls on soil microbial community activity and composition**"

Nicholas C. Dove[1,2]*, Morgan E. Barnes[1,3], Kimber Moreland[1,4], Robert C. Graham[5], Asmeret Asefaw Berhe[6,7], and Stephen C. Hart[6,7]

[1]Environmental Systems Graduate Group, University of California, Merced, CA 95343, USA

[2]Biosciences Division, Oak Ridge National Laboratory, Oak Ridge, TN 37830, USA

[3]Ecosystem Science, Pacific Northwest National Laboratory, Richland, WA 99352, USA

[4]Center for Accelerator Mass Spectrometry, Lawrence Livermore National Laboratory, Livermore, CA, USA

[5]Department of Environmental Sciences, University of California, Riverside, CA, USA

[6]Department of Life & Environmental Sciences, University of California, Merced, CA 95343, USA

[7]Sierra Nevada Research Institute, Merced, CA 95343, USA

*Corresponding author: Nicholas C. Dove (ndove7@gmail.com); phone: +1-508-277-5039

SUPPLEMENTAL TABLES

Table S1: Study site characteristics.

Site	Pseudonym[1]	Dominant vegetation	Soil taxonomic family	MAT[2] (C)	MAP[3] (cm)	Maximum sampling depth (cm)
Oak Savanna (OS)	San Joaquin Experimental Grassland	*Quercus douglasii*	Coarse loamy, mixed, active, thermic Mollic Haploxeralfs	16.7	50.2	270
Pine Oak Forest (POF)	Soaproot Saddle	*Calocedrus decurrens*, *Pinus ponderosa*	Fine loamy, mixed, semiactive, mesic Ultic Haploxeralfs	13.6	86	232
Mixed Conifer Forest (MCF)	Providence Creek Watershed	*Pinus ponderosa*, *Abies concolor*	*Coarse loamy, mixed, superactive, frigid Humic Dystroxerepts*	9.2	99.4	216
Subalpine Forest (SAF)	Shorthair	*Abies magnifica, Pinus contorta*	Sandy-skeletal, mixed, Typic Cryorthents	4.8	107	115
Sagerbush Shrubland (SBS)	Granite 2 (GR2)	*Artemisia tridentata* ssp. vaseyana	Coarse-loamy, mixed, superactive, mesic Typic Calciargids	8.5	24.7	80
Pinyon Juniper Woodland (PJW)	Granite 3 (GR3)	*Pinus monophylla, Juniperus occidentalis, Artemisia tridentata ssp. vaseyana*	Coarse-loamy, mixed, superactive, mesic Typic Calciargids	7.3	27.7	55
Subalpine Shrubland (SAS)	Granite 5 (GR5)	*Pinus monophylla, Juniperus occidentalis, Artemisia tridentata ssp. vaseyana*	Loamy-skeletal, mixed, superactive, mesic Typic Haplargids	3.3	42.1	53
Alpine Desert Grassland (ADG)	Barcroft	*Koeleria macrantha, Carex douglasii*	Loamy-skeletal, mixed, superactive Typic Haplocryids	0.1	53.8	172

[1] Pseudonyms refer to previously published site names.

[2] Mean annual air temperature (1981 – 2018, PRISM Climate Group, Oregon State University, http://prism.oregonstate.edu; accessed May 2020).

[3] Mean annual precipitation (1981 – 2018, PRISM Climate Group, Oregon State University, http://prism.oregonstate.edu; accessed May 2020).

Table S2: Horizons and depths in each soil profile across sites (provided in a separate data sheet).

Table S3: χ^2 values of competing mixed effects models of enzyme activity (μmol) normalized by soil weight, microbial biomass carbon (MBC), and soil organic carbon (SOC). Bolded coefficients represent significant (p < 0.05) fixed effects. Key: BG = β-glucosidase, NAG = N-acetylglucosamine, AP = acid phosphatase, MAT = mean annual temperature (°C), MAP = mean annual precipitation (mm).

	Enzyme	Model*	Horizon	MAT	Horizon*MAT	MAP	Horizon*MAP
μmol kg^{-1} soil	BG	1	**1.39E+01**				
		2	**1.54E+01**	2.89E+00	**1.68E+01**		
		3	**1.41E+01**			3.07E-02	**6.55E+00**
		4	**1.53E+01**	**4.14E+00**	**1.27E+01**	6.26E-02	4.11E+00
	NAG	1	4.76E+00				
		2	5.55E+00	1.52E-02	**2.10E+01**		
		3	5.41E+00			1.16E+00	**7.45E+00**
		4	**6.04E+00**	3.00E-04	**1.94E+01**	2.32E+00	5.53E+00
	AP	1	**2.06E+01**				
		2	**2.06E+01**	2.60E-01	2.87E+00		
		3	**2.15E+01**			2.85E-02	**9.25E+00**
		4	**2.14E+01**	1.21E-01	1.67E+00	8.17E-02	**7.68E+00**
μmol mol^{-1} MBC	BG	1	5.20E+00				
		2	**6.02E+00**	9.99E-02	**6.58E+00**		
		3	5.52E+00			**4.01E+00**	9.40E-02
		4	**6.01E+00**	3.30E-03	**7.64E+00**	**5.29E+00**	6.55E-02
	NAG	1	3.53E+00				
		2	4.67E+00	8.82E-01	**1.05E+01**		
		3	4.29E+00			**5.22E+00**	8.72E-01
		4	5.69E+00	1.35E+00	**1.16E+01**	**7.20E+00**	-1.89E-05
	AP	1	**7.20E+00**				
		2	**8.82E+00**	**5.72E+00**	1.26E+00		
		3	**6.73E+00**			**5.70E+00**	6.24E-01
		4	**8.14E+00**	**4.13E+00**	1.13E+00	**4.28E+00**	5.03E-01
μmol mol^{-1} SOC	BG	1	**2.47E+01**				
		2	**2.72E+01**	1.30E-01	**1.69E+01**		
		3	**2.66E+01**			3.24E+00	**5.74E+00**
		4	**2.86E+01**	1.16E-01	**1.32E+01**	2.56E+00	2.88E+00

NAG	1	**1.61E+01**				
	2	**1.88E+01**	**7.25E+00**	**1.98E+01**		
	3	**1.69E+01**			5.86E-01	**6.20E+00**
	4	**2.00E+01**	**7.74E+00**	**1.88E+01**	4.06E-01	**6.74E+00**
AP	1	**4.34E+01**				
	2	**4.23E+01**	**7.56E+00**	1.66E+00		
	3	**4.50E+01**			1.77E+00	5.05E+00
	4	**4.46E+01**	**1.01E+01**	8.81E-01	3.81E+00	4.41E+00

*1 = $\log_{10}$(enzyme) ~ Horizon + (1|Pit)
2 = $\log_{10}$(enzyme) ~ Horizon + MAT + Horizon *MAT + (1|Pit)
3 = $\log_{10}$(enzyme) ~ Horizon + MAP + Horizon *MAP + (1|Pit)
4 = $\log_{10}$(enzyme) ~ Horizon + MAT + Horizon *MAT + MAP + Horizon *MAP + (1|Pit).

Table S4: Beta coefficients and *p*-values of beta-regressions of the relative abundance (%) of ectomycorrhizal (EM) and arbuscular mycorrhizal (AM) fungi with soil depth (cm). Bolded values represent sites where depth is significant ($p < 0.05$).

		EM		AM	
	Site	β	*p*	β	*p*
SSCZO	OS	< |0.01|	0.950	< |0.01|	0.918
	POF	- 0.08	0.059	< |0.01|	0.596
	MCF	0.02	0.740	--	--
	SAF	**0.18**	**0.004**	< |0.01|	0.985
WMET	SBS	- 0.11	0.360	**- 0.13**	**0.033**
	PJW	0.25	0.205	- 0.02	0.578
	SAS	< |0.01|	0.962	- 0.03	0.760
	ADG	- 0.02	0.728	**- 0.04**	**0.030**

SUPPLEMENTAL FIGURES

Figure S1: Principal coordinates analysis (PCoA) plots of prokaryote (**A**) and fungal (**B**) community composition across study sites. The percentage in parentheses indicates the variation explained by each axis.

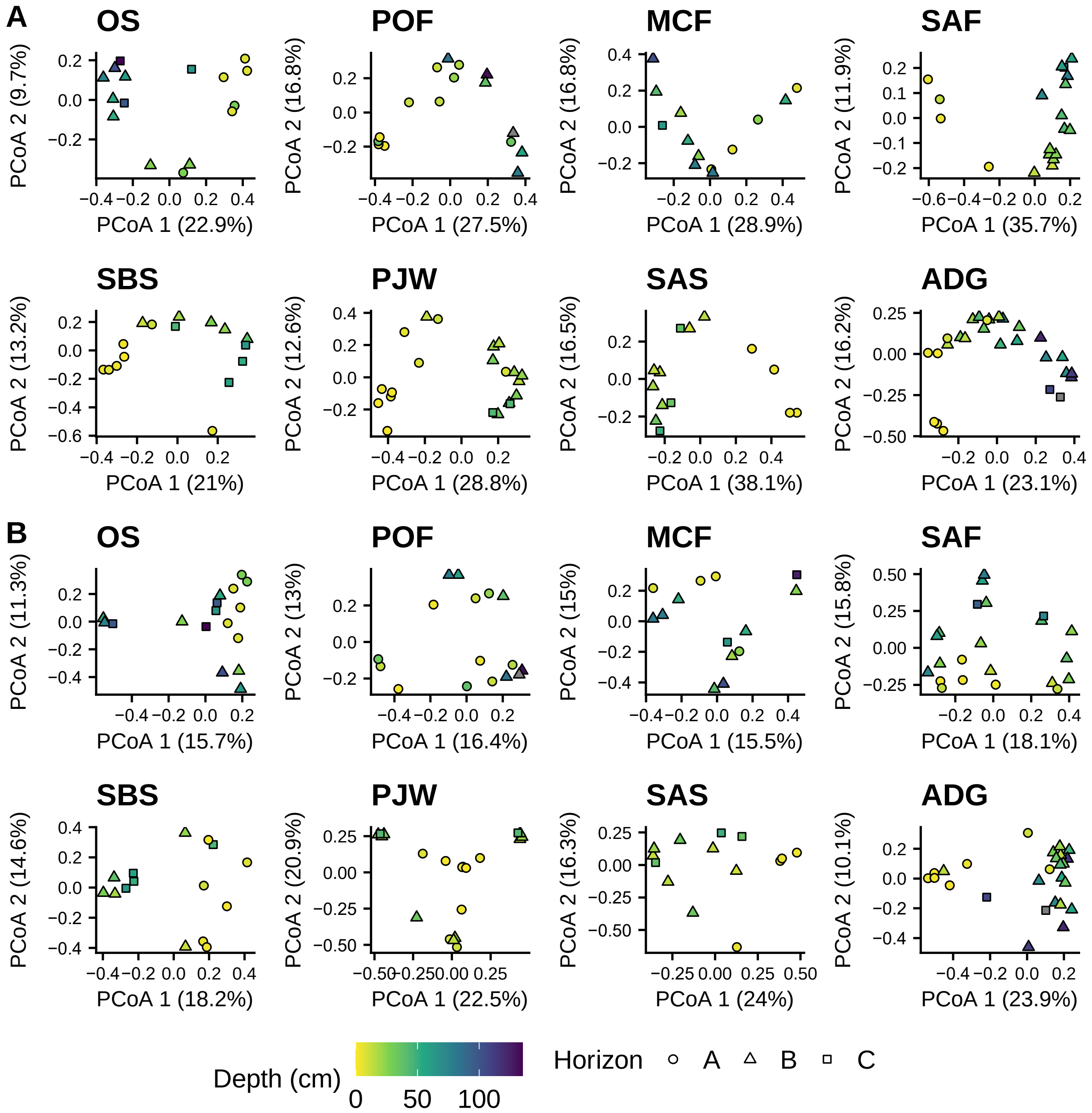

Figure S2: Mean and standard error of within horizon β-diversity (using Bray-Curtis dissimilarity).

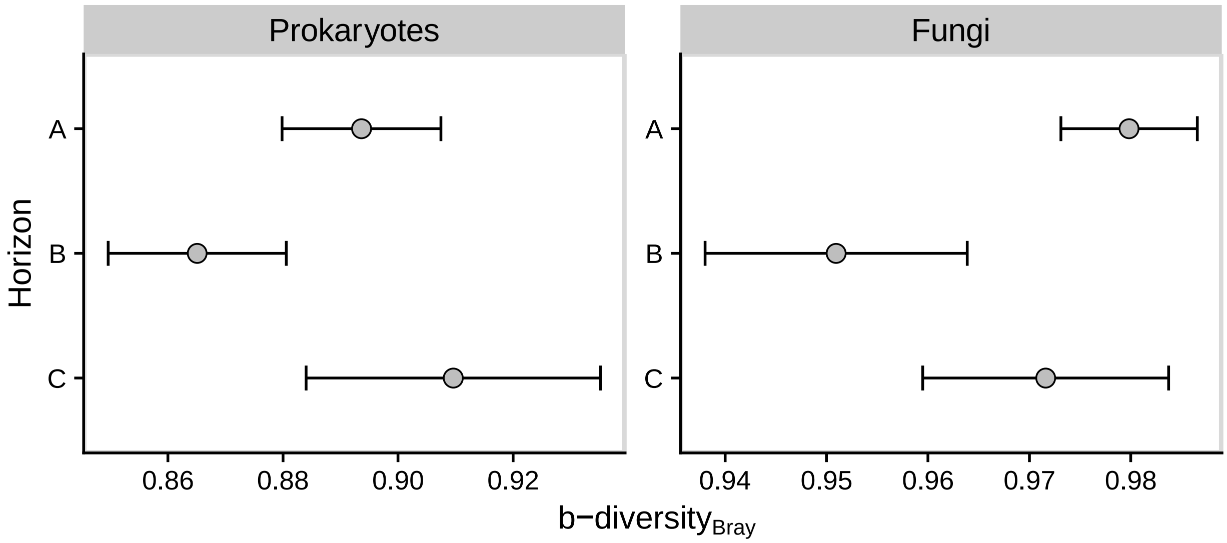

Figure S3: Extracellular enzyme activity of β-glucosidase (BG), N-acetylglucosamine (NAG), acid phosphatase (AP) on soil weight basis colored by site (**A**), mol microbial biomass carbon (MBC, **B**), and mol soil organic carbon (SOC, **C**).

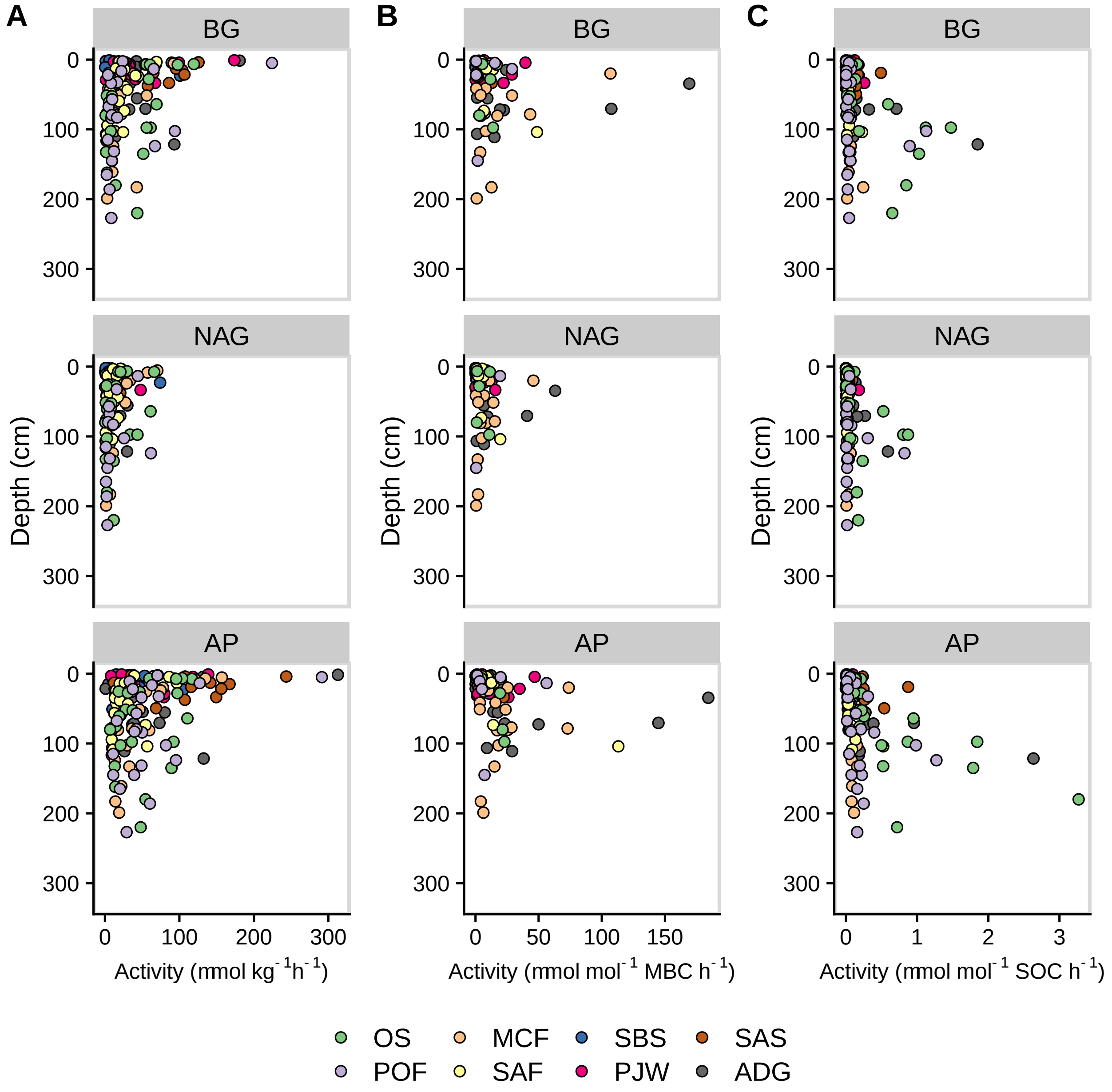

Figure S4: Extracellular enzyme activity of β-glucosidase (BG), N-acetylglucosamine (NAG), acid phosphatase (AP) on soil weight basis across sites and master horizons. Points represent samples and boxplots represents the median and interquartile range. Whiskers extend to the highest/lowest value within 1.5 times the interquartile range from the interquartile range.

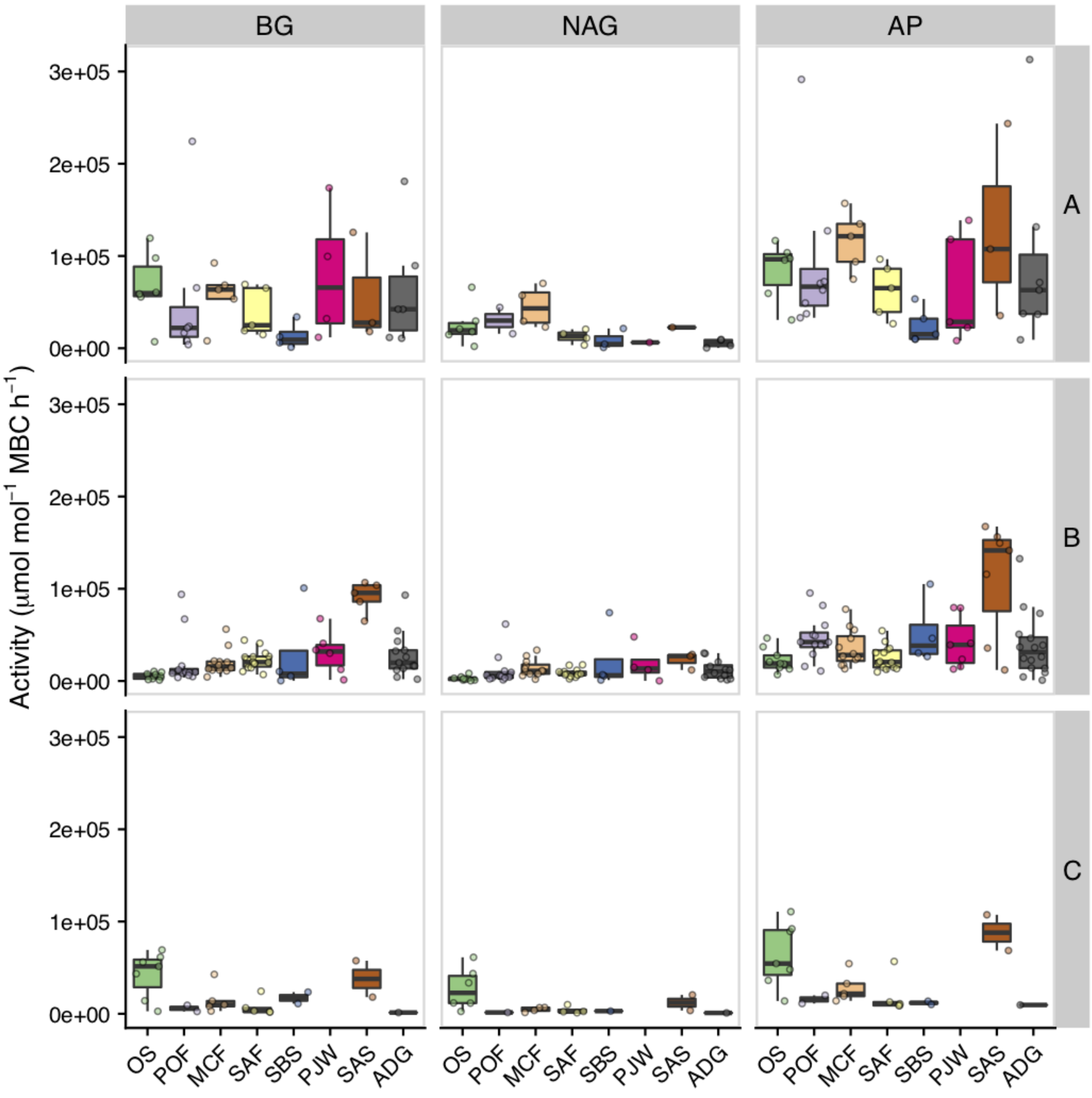

Figure S5: Extracellular enzyme activity of β-glucosidase (BG), N-acetylglucosamine (NAG), acid phosphatase (AP) on a mol microbial biomass carbon (MBC) basis across sites and master horizons. Points represent samples and boxplots represents the median and interquartile range. Whiskers extend to the highest/lowest value within 1.5 times the interquartile range from the interquartile range.

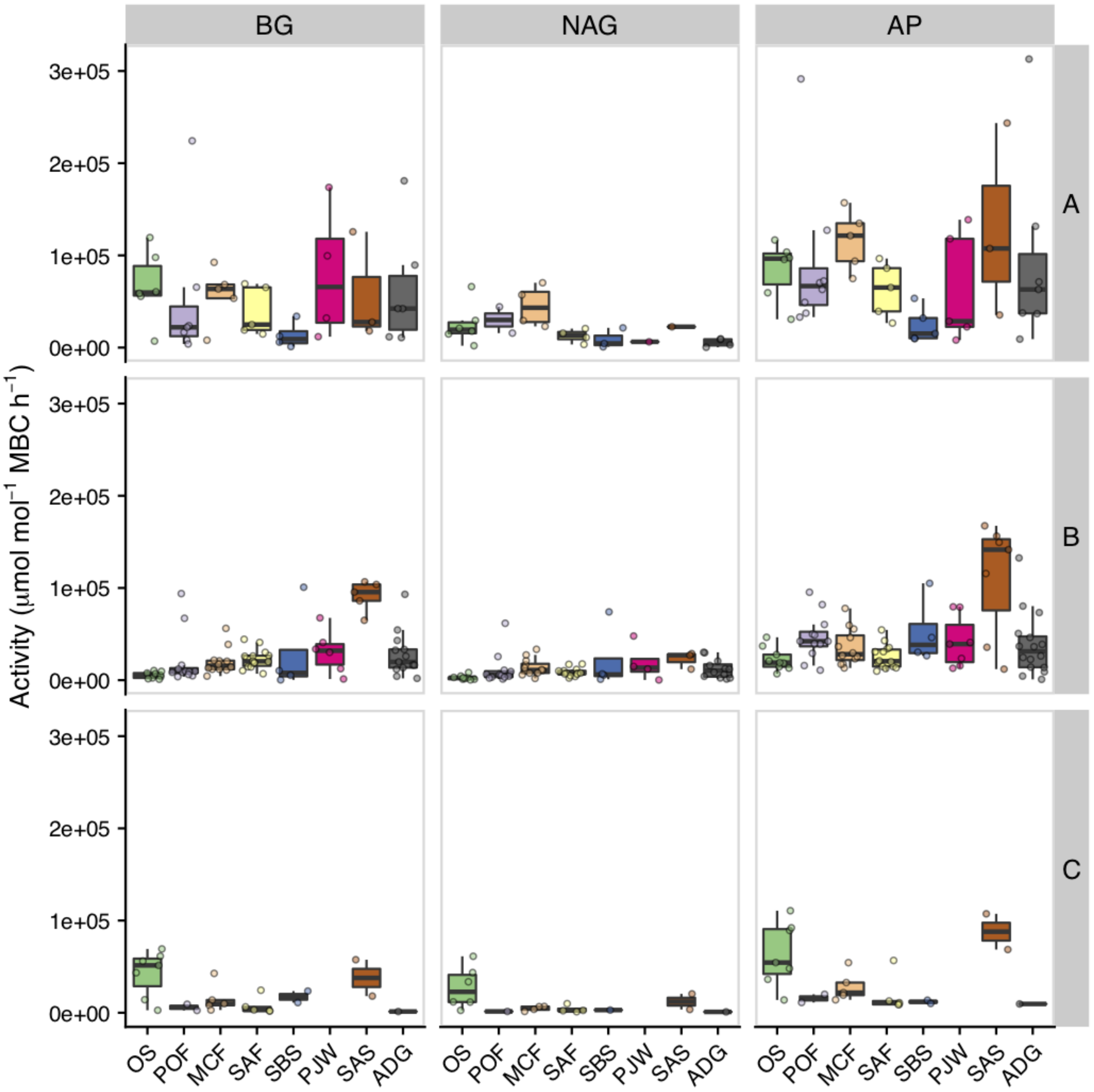

Figure S6: Extracellular enzyme activity of β-glucosidase (BG), N-acetylglucosamine (NAG), acid phosphatase (AP) on a mol soil organic carbon (SOC) basis across sites and master horizons. Points represent samples and boxplots represents the median and interquartile range. Whiskers extend to the highest/lowest value within 1.5 times the interquartile range from the interquartile range.

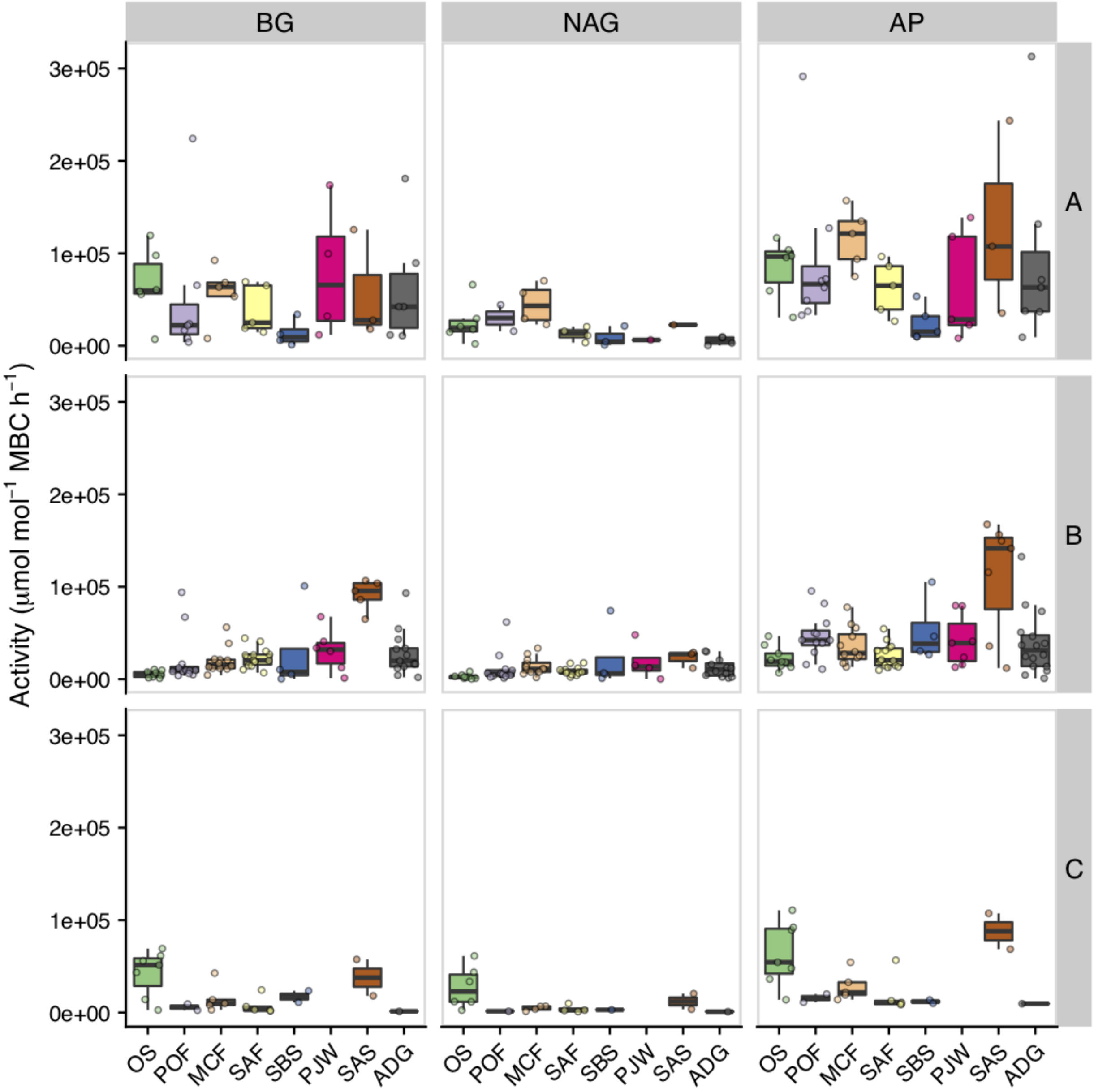

Figure S7: Average relative abundance of prokaryotic taxa (**A**) and fungal classes (**B**) that significantly change (p-adjusted < 0.05) with depth at each site. Colored lines are calculated using loess regression of average relative abundance at each site (n = 4). Dashed lines represent depths of master horizon boundaries (boundary between B and C horizons is not shown when a C horizon was not encountered).

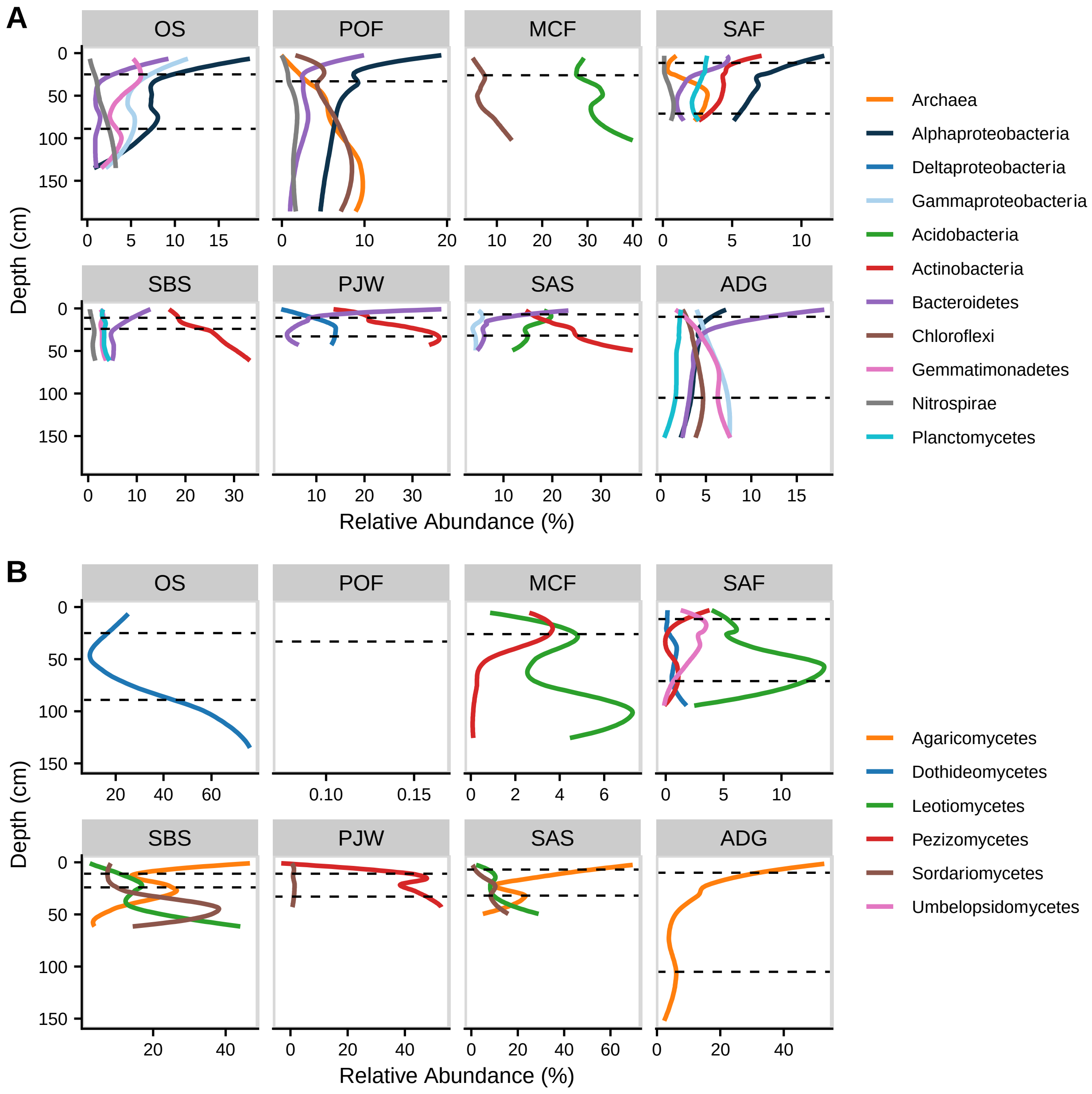

Figure S8: Relative abundance of ectomycorrhizal (**A**) and arbuscular mycorrhizal (**B**) fungi among sites and master horizons. Bars represent means, error bars represent standard error of the mean, and points represent samples. Letters represent significant differences among sites for each master horizon (Tukey HSD, $p < 0.05$).

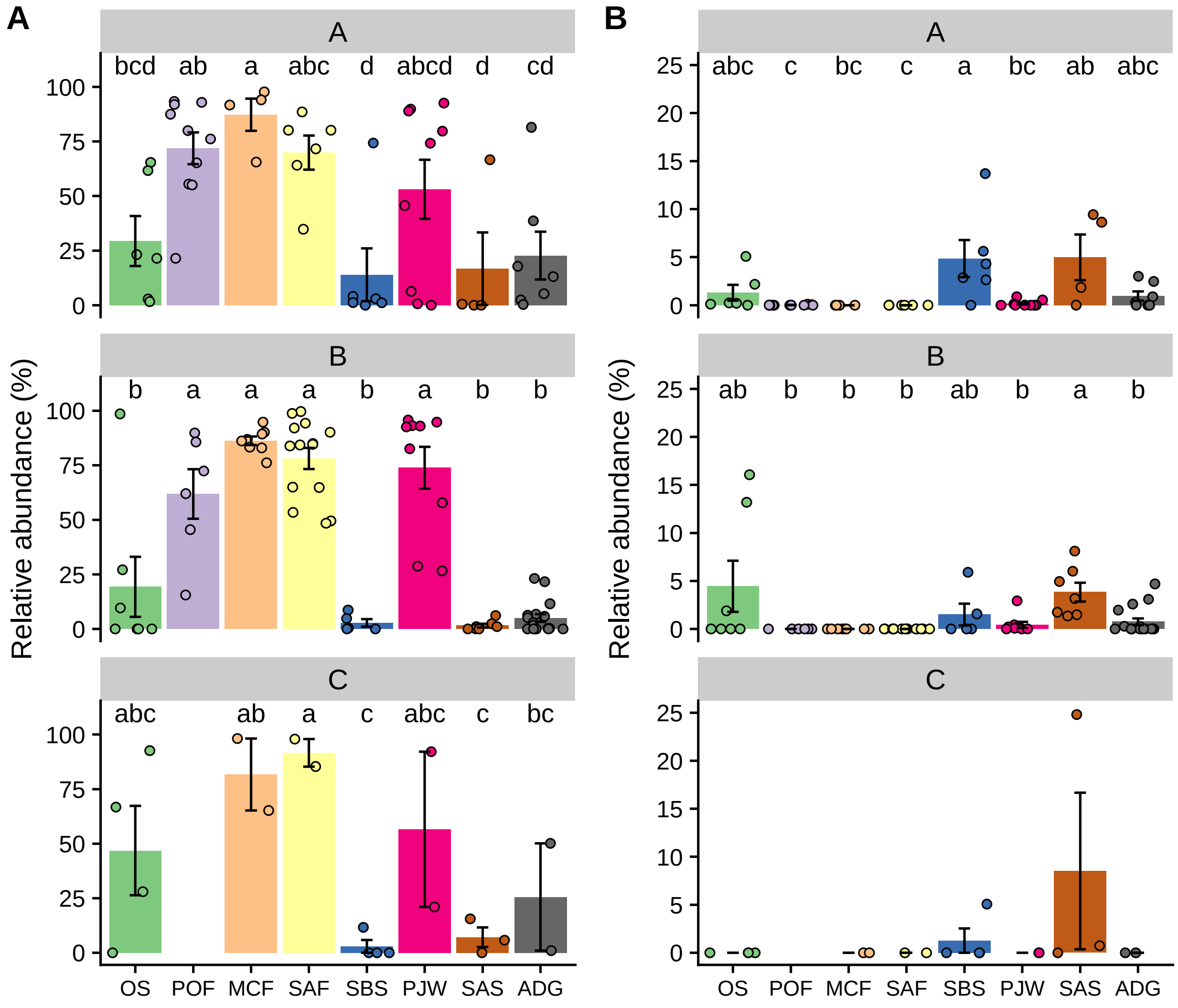

Supplement: Supplementary file 1 — Supplementary Material [file 43705_2021_81_MOESM1_ESM.pdf]
